# The Intersection of Aging, Longevity Pathways, and Learning and Memory in *C. elegans*

**DOI:** 10.3389/fgene.2012.00259

**Published:** 2012-11-26

**Authors:** Geneva M. Stein, Coleen T. Murphy

**Affiliations:** ^1^Glenn Laboratories for Aging Research, Department of Molecular Biology, Lewis-Sigler Institute for Integrative Genomics, Princeton UniversityPrinceton, NJ, USA

**Keywords:** aging, *C. elegans*, insulin signaling, memory, learning, longevity, behavior, neurons

## Abstract

Our understanding of the molecular and genetic regulation of aging and longevity has been greatly augmented through studies using the small model system, *C. elegans*. It is important to test whether mutations that result in a longer life span also extend the health span of the organism, rather than simply prolonging an aged state. *C. elegans* can learn and remember both associated and non-associated stimuli, and many of these learning and memory paradigms are subject to regulation by longevity pathways. One of the more distressing results of aging is cognitive decline, and while no gross physical defects in *C. elegans* sensory neurons have been identified, the organism does lose the ability to perform both simple and complex learned behaviors with age. Here we review what is known about the effects of longevity pathways and the decline of these complex learned behaviors with age, and we highlight outstanding questions in the field.

## Introduction

Human learning and memory decline with age. Understanding the genetic basis of this decline could lead to preventative treatments and therapies. *C. elegans* is an ideal model organism to identify genetic pathways that regulate both aging and cognitive decline, with its established use as a model for aging (Klass, 1977; Friedman and Johnson, 1988; Hosono et al., 1989; Kenyon et al., 1993), synapse formation and function (Sulston and Horvitz, 1977; Lewis et al., 1980; Sulston et al., 1983; Rand and Russell, 1984; White et al., 1986), and neuron-mediated behaviors (Ward, 1973; Dusenbery, 1974; Chalfie and Sulston, 1981; Avery and Horvitz, 1989; Bargmann and Horvitz, 1991).

*C. elegans* is a small (1 mm long) transparent nematode found worldwide in rotting vegetable matter (Brenner, 1974; Felix and Braendle, 2010). The cell lineages of all 959 somatic cells in the adult hermaphrodite have been mapped (Sulston and Horvitz, 1977; Kimble and Hirsh, 1979), as has the position and connectivity of the 302 neurons (White et al., 1986; Varshney et al., 2011). Additionally, more than 80% of *C. elegans* genes have a human ortholog (Lai et al., 2000). *C. elegans* is a well-established model for studying the genetic basis of aging. While the normal lifespan of *C. elegans* is 23 weeks, many lifespan-extending mutants have been identified. Insulin/IGF-1 signaling (IIS) and caloric restriction (CR) regulate aging in *C. elegans*, and are conserved in higher organisms (McCay and Crowell, 1934; Kenyon et al., 1993; Lakowski and Hekimi, 1998; Bluher et al., 2003; Wood et al., 2004; Suh et al., 2008; Anderson et al., 2009). Because of its simple nervous system, *C. elegans* is also a model for synapse function (reviewed in Richmond, 2007), neuron-mediated behaviors (reviewed in Hobert, 2003), and learning and memory (reviewed in Ardiel and Rankin, 2010). Here we review age-related changes in learning and memory and their regulation by insulin signaling, mitochondrial metabolism, and CR.

## *C. elegans* Longevity Pathways

### Insulin/IGF-1 signaling

Reduction of insulin signaling was first found to increase longevity in *C. elegans* (Kenyon et al., 1993), and this evolutionarily conserved pathway has also been shown to influence lifespan in flies, mice, and humans (Clancy et al., 2001; Tatar et al., 2001; Bluher et al., 2003; Suh et al., 2008). In worms, there is a single insulin receptor tyrosine kinase homolog, *daf-2* (Kimura et al., 1997), which was originally discovered for its role in the formation of dauers (Riddle et al., 1981), an alternative developmental stage that *C. elegans* can enter in order to survive in harsh environments. *daf-2* Loss-of-function mutants have twice the lifespan of wild-type worms (Kenyon et al., 1993). The lifespan extension observed in *daf-2* worms is dependent on the forkhead box O (FOXO) protein transcription factor, DAF-16 (Kenyon et al., 1993; Ogg et al., 1997; Lin et al., 2001). When the insulin-like growth factor 1 receptor (IGFR) DAF-2 is activated, a PI3 kinase cascade is triggered that ultimately phosphorylates DAF-16/FOXO and sequesters the transcription factor in the cytoplasm (Lin et al., 2001). *age-1*, The first gene discovered to regulate longevity in *C. elegans* (Friedman and Johnson, 1988), encodes an ortholog of the p110 catalytic subunit of Class IA phosphoinositide 3-kinase (PI3K) (Morris et al., 1996). The longevity of *age-1* mutants is dependent on *daf-16* (Dorman et al., 1995), and genetic analysis showed that AGE-1/PI3K functions downstream of DAF-2/IGFR and upstream of the AKT-2/AKT-1 and PDK-1 kinases and DAF-16/FOXO transcription factor (Paradis and Ruvkun, 1998; Paradis et al., 1999). In the absence of insulin signaling, DAF-16/FOXO is localized to the nucleus, where it regulates a host of genes that promote longevity and stress resistance. The proteins involved in IIS and many of the downstream targets of DAF-16/FOXO that contribute to the phenotypic outputs of insulin signaling (Murphy et al., 2003) have been identified and characterized.

*C. elegans* encodes approximately 40 insulin-like peptides that can act as either DAF-2 agonists and antagonists (Pierce et al., 2001; Li et al., 2003; Murphy et al., 2003, 2007). Most of the insulin-like peptides are expressed in the neurons, though a few are expressed in the intestine as well (Pierce et al., 2001; Li et al., 2003; Murphy et al., 2007). Pierce et al. (2001) identified INS-1 as the closest homolog of human insulin, and found that overexpression of *ins-1* or human insulin antagonizes *daf-2* signaling, moderately increasing lifespan and enhancing dauer arrest. Loss of *ins-1* did not influence lifespan or dauer entry, probably due to functional redundancy among insulin-like peptides (Pierce et al., 2001). Along with influencing dauer arrest and longevity, *ins-1* has recently been implicated in regulation of many neuron-specific sensory behaviors, such as neuropeptide feedback, serotonergic signaling, and starvation-associated aversion learning (Kodama et al., 2006; Tomioka et al., 2006; Chalasani et al., 2010; Lin et al., 2010; Harris et al., 2011).

### Mitochondrial metabolism

Through metabolic processes, mitochondria produce the most reactive oxygen species (ROS) in a majority of eukaryotic cells (Kowaltowski et al., 2009). Excessive ROS react with proteins, DNA, RNA, and lipids, causing oxidative damage (Richter et al., 1988; Grune et al., 1997; Crawford et al., 1998). (Details of the specific mechanism of ROS production are reviewed in Kowaltowski et al., 2009). Oxidative damage caused by ROS is thought to contribute to aging, though the extent and mechanism of its action is not yet known. Mutations in the *C. elegans* *clk-1* gene, which encodes a hydroxylase in the electron transport chain that is required for ubiquinone biosynthesis (Miyadera et al., 2001), result in extended lifespan (Wong et al., 1995). *isp-1* Encodes the iron sulfate protein of the electron transport chain, and mutation of *isp-1* decreases metabolic respiration and increases lifespan (Feng et al., 2001). Mutations in two additional genes in the electron transport chain, *mev-1*, which encodes a cytochrome *b* homolog, and *gas-1*, which encodes the major 49 kDa iron protein subunit of complex 1, show reduced resistance to ROS and shortened lifespan (Adachi et al., 1998; Ishii et al., 1998; Kayser et al., 2001, 2004). RNAi screens have identified many mitochondrial genes that regulate lifespan in a *daf-16* and *daf-2-*independent manner (Dillin et al., 2002; Lee et al., 2003). While the exact mechanisms by which metabolic mutations influence longevity remain largely unknown, lower levels of ROS-damaged proteins in *clk-1* mutants and higher levels in *gas-1* mutants support the theory that damage caused by ROS contributes to functional decline during aging (Kayser et al., 2004).

### Caloric restriction

Restricting caloric intake to 60–70% of normal levels was first shown to extend rat lifespan by McCay et al. (1935) and has since been demonstrated in many organisms, from yeast to primates (McCay et al., 1935; Weindruch, 1996; Lin et al., 2000). Many methods of calorically restricting wild-type *C. elegans* result in a lengthened lifespan, including growing worms in or on diluted bacteria (BDR) or in axenic media (ADR) (Klass, 1977; Hosono et al., 1989; Greer et al., 2007). Calorically restricting *daf-2* or *daf-16* mutants using BDR or ADR extends lifespan compared to *daf-2* or *daf-16* alone, suggesting that CR regulates aging independent of insulin signaling (Houthoofd et al., 2003; Crawford et al., 2007). Pharyngeal pumping mutants (e.g., *eat-2*) extend longevity due to defects in feeding (Raizen et al., 1995; Lakowski and Hekimi, 1998). Like direct CR, *eat-2* regulates lifespan independent of insulin signaling, as *eat-2;daf-2* double mutants live 20% longer than *daf-2* alone (Crawford et al., 2007). The PHA-4/FOXA1 transcription factor regulates CR-mediated longevity independently of its essential role in pharyngeal development (Panowski et al., 2007). Reducing *pha-4* expression does not suppress the longevity of *daf-2* or *isp-1* mutants (Panowski et al., 2007), but PHA-4 activity is required for the lifespan extension of germline-less mutants (Hansen et al., 2008; Lapierre et al., 2011), suggesting that the germline and CR pathways may converge but that they are independent of IIS and mitochondrial longevity pathways. Adult-specific expression of *pha-4* is required for the extended longevity of BDR-treated worms and of *eat-2* mutants. The full complement of molecular mechanisms required for CR-mediated longevity downstream of PHA-4/FOXA1 activity are not yet known.

## Sensory Systems in *C. elegans*

### Behavior

Despite the simplicity of this invertebrate system, the *C. elegans* model permits the analysis of both simple and complex behaviors at the individual gene level (reviewed in Hobert, 2003; de Bono and Maricq, 2005). *C. elegans* can sense and respond to temperature changes, gentle and harsh touch, O_2_ and CO_2_ concentration, and osmolarity, and can taste soluble chemicals and smell volatile odors (Ward, 1973; Dusenbery, 1974; Hedgecock and Russell, 1975; Culotti and Russell, 1978; Chalfie and Sulston, 1981; Bargmann et al., 1990; Bargmann and Horvitz, 1991; Gray et al., 2004; Bretscher et al., 2008; Hallem and Sternberg, 2008). Worms respond to these sensations by changing their normal locomotory behavior of smooth forward movement and turns (Croll, 1975; Niebur and Erdos, 1991) to instead chemotax using biased random walk and weathervane mechanisms (Pierce-Shimomura et al., 1999; Iino and Yoshida, 2009). *C. elegans* reverse in response to negative stimuli, suppress turns in response to attractive stimuli, adjust their speed and rate of body bends, and combine multiple behaviors to respond to more complex sensory environments (reviewed in Mori, 1999; Hobert, 2003; Bargmann, 2006; Goodman, 2006).

*C. elegans* integrate sensory stimuli and exhibit behavioral plasticity. Well-characterized forms of non-associative behavioral plasticity include adaptation to inherently attractive odors (Colbert and Bargmann, 1995) and habituation in response to multiple taps (Rankin et al., 1990). Associative behaviors include the ability to associate feeding state with temperature (thermotaxis) (Hedgecock and Russell, 1975; Mohri et al., 2005), salt concentration (salt learning) (Saeki et al., 2001), odor (olfactory learning) (Nuttley et al., 2002; Zhang et al., 2005; Torayama et al., 2007; Ha et al., 2010; Kauffman et al., 2010), and pathogenic state (Zhang et al., 2005). In addition to both associative and non-associative learning, *C. elegans* is able to form both short-term and long-term memories, lasting as long as 24 h (Rankin et al., 1990; Colbert and Bargmann, 1995; Gomez et al., 2001; Tomioka et al., 2006; Kano et al., 2008; Kauffman et al., 2010). While these behavioral phenotypes, as well as many of the genes regulating chemotaxis, thermotaxis, salt learning, adaptation, and habituation have been studied, very little is known about the specific molecular mechanisms involved in integrating multiple sensory signals (reviewed in de Bono and Maricq, 2005; Ardiel and Rankin, 2010).

### Sensory neurons

*C. elegans* has a simple nervous system that contains only 302 neurons. The positions of these neurons and their processes have been mapped and are highly stereotyped between individuals (White et al., 1986; Varshney et al., 2011). GFP fusions have been used to identify neuron-specific gene expression and protein localization in the transparent *C. elegans* (Chalfie et al., 1994; Nonet, 1999). Although electrophysiological techniques have been used to study the activity of specific neurons (Goodman et al., 1998; Lockery and Goodman, 1998; Richmond and Jorgensen, 1999), genetically encoded calcium indicators, such as Cameleon (Miyawaki et al., 1997; Kerr et al., 2000) and more recently, GCaMP (Nakai et al., 2001; Chronis et al., 2007; Tian et al., 2009), have allowed the measurement of neuronal activity in live, behaving worms, and the advent of microfluidic techniques has allowed the assessment of neuronal activity in response to stimuli (Suzuki et al., 2003; Chalasani et al., 2007; Chronis et al., 2007).

*C. elegans* has the ability to sense certain stimuli using only a single neuron or a subset of neurons. These sensory neurons communicate through interneurons and command interneurons to regulate motor neuron output and motor response to stimuli (reviewed in Hobert, 2003). Many neurons required to detect sensory stimuli have been identified, including those involved in odortaxis (AWA, AWB, AWC, ASH, and ADL), chemotaxis (ASE, ASK, ADF, ASG, and ASI), touch response (ALM, AVM, PVM, IL1, and OLQ) as well as many others involved in mechanosensory response to stimuli (reviewed in Bargmann and Kaplan, 1998) and thermotaxis (AFD, AWC, and ASI) (Mori and Ohshima, 1995; Biron et al., 2008; Kuhara et al., 2008; Beverly et al., 2011). The involvement of interneurons to mediate sensory output and integration is not as well understood, although circuits for thermotaxis, touch response, and chemotaxis that include interneurons such as AIA and AIY have been characterized using the original White et al. (1986) wiring diagram coupled with neuron ablation (Chalfie et al., 1985; Mori and Ohshima, 1995; Bargmann and Kaplan, 1998; Zheng et al., 1999; Tsalik and Hobert, 2003; Gray et al., 2005). Cell-specific genetic rescue (Mello et al., 1991), *in vivo* calcium imaging (Kerr et al., 2000; Suzuki et al., 2003; Chronis et al., 2007; Tian et al., 2009), electrophysiology (Goodman et al., 1998; Lockery and Goodman, 1998; Richmond and Jorgensen, 1999; Richmond et al., 1999), and genomic techniques (Wenick and Hobert, 2004) have been used to verify and refine these circuit models, which can then be used as a starting point when testing the neurons involved in specific behaviors.

### The aging neuron

Until recently, it was thought that *C. elegans* neurons did not show age-related morphological decline at either a cellular or subcellular level, because while other tissues, such as skin and muscle, deteriorate with age (Garigan et al., 2002), neurons remained surprisingly intact (Herndon et al., 2002). These data seem counterintuitive, considering multiple sensory behaviors as well as motility decline with age in *C. elegans* (Glenn et al., 2004; Murakami et al., 2005; Hsu et al., 2009; Kauffman et al., 2010; Guo et al., 2012) and changes in dendritic spines and synapse number with age have been observed in other organisms, including non-human primates and rats (reviewed in Burke and Barnes, 2006; Morrison and Baxter, 2012). Due to this incongruity, recent work has again tested the integrity of neurons and found that while neuronal cell bodies stay intact, neuronal processes, subcellular structures (Pan et al., 2011; Tank et al., 2011; Toth et al., 2012), and neuronal activity (Chokshi et al., 2010; Mulcahy et al., 2012) all show age-dependent changes.

Neuronal aging is associated with morphological changes that include ectopic neurite branching from the soma and processes, GFP beading within the process, and blebbing that results in a “wavy” process (Pan et al., 2011; Tank et al., 2011; Toth et al., 2012). Blebbing may be the precursor to neurite formation (Pan et al., 2011). The extent and type of abnormal cellular structures in aged animals is highly variable across neurons (Pan et al., 2011; Tank et al., 2011; Toth et al., 2012). Excess ectopic neurites are correlated with a decrease in gentle touch response and mobility (Tank et al., 2011), and neurite branching occurs as early as day 8 (Pan et al., 2011). While these experiments analyzed touch neurons and motor neurons that run along the *C. elegans* mid-section, Toth et al. (2012) found through analysis of EM images that synapses in the nerve ring and ventral ganglion are depleted of vesicles in day 15 animals. Older *daf-2* worms have many fewer neuronal abnormalities with age than do similarly aged wild-type worms (Pan et al., 2011; Tank et al., 2011; Toth et al., 2012). The *daf-2* slowed morphology change phenotype is dependent on *daf-16* (Pan et al., 2011; Toth et al., 2012), and *daf-16* mutants have increased neurite branching with age compared to wild-type (Pan et al., 2011; Tank et al., 2011). Tank et al. (2011) found that neuron-specific rescue of *daf-16* in *daf-16;daf-2* double mutants restored the *daf-2* phenotype in mechanosensory neurons. Although Pan et al. (2011) found excessive neurite branching in *daf-16* mutants, they could not rescue this phenotype with neuron-specific *daf-16* expression. Thus, it is unclear whether DAF-16 acts cell autonomously or non-autonomously to regulate neurite morphology with age.

The heat-shock transcription factor HSF-1 is required for *daf-2*-mediated longevity, and functions with *daf-16* to promote proteostasis and mediate longevity (Garigan et al., 2002; Hsu et al., 2003; Morley and Morimoto, 2004; Cohen et al., 2006). *hsf-1* Mutants have significant increases in all age-related morphological changes in neurons (Pan et al., 2011; Toth et al., 2012). In addition to an excess of normal age-related changes, *hsf-1* mutants also have breaks in their neuronal processes (Toth et al., 2012). *hsf-1;daf-16* Double mutants do not have more neuronal defects than single mutants and therefore, probably act in the same pathway to regulate neuronal aging (Pan et al., 2011).

Unlike insulin signaling mutants, *eat-2* mutants have a long lifespan, but have normal rates of neurite branching with age (Tank et al., 2011). *clk-1* Mutants have a phenotype that is similar to *daf-2* mutants, suppressing neurite outgrowth in older worms (Tank et al., 2011). Together, these data show that neuron morphology does change with age and is regulated by specific longevity pathways. Given that morphological defects such as abnormal branch formation are regulated during development and also arise with age, it would be interesting to test if pathways required for neuron development have an additional role in the regulation of neuron maintenance with age (Benard and Hobert, 2009).

Morphological changes in sensory neurons have not yet been reported. However, using microfluidics, Chokshi et al. (2010) found that the ASH sensory neuron’s calcium response to glycerol changed with age. Specifically, day 1 adults had a lower peak response to glycerol than did days 3 or 4 adults, perhaps correlating with the peak reproductive period, but day 5 adults had a much smaller peak response than days 1–4. Chokshi et al. (2010) also identified oscillations in the calcium response to glycerol exposure of day 1 adult worms that were not present in days 3, 4, and 5 adult worms. The calcium responses of older *C. elegans* and/or other sensory neurons have not yet been investigated. Interestingly, mutants with defects in sensory cilia and worms with specific sensory neurons ablated are long-lived, suggesting that *C. elegans* lifespan is regulated by perception of its environment (Apfeld and Kenyon, 1999; Alcedo and Kenyon, 2004). Sensory mutant lifespan can be partially rescued in a *daf-16* background, showing that extension is regulated in part by insulin signaling (Apfeld and Kenyon, 1999; Alcedo and Kenyon, 2004).

## Learning and Memory Paradigms

Associative learning and memory are acquired with training that pairs a conditioned stimulus with an unconditioned stimulus, known as “classical conditioning.” First made famous by Pavlov’s (1927) original experiment training dogs to associate food with a ringing bell, classical conditioning has been tested in organisms from *Drosophila* to mice, rats, and humans, and more recently, *C. elegans*. Many different classical conditioning paradigms have been used in human experiments, ranging from the original controversial Little Albert experiment in which a baby was trained to associate a white rat with a loud noise (Watson and Raynor, 1920), to more recent experiments associating neutral tones with harsh white noise (Hensman et al., 1991). Paradigms in model systems include training *Drosophila*, mice, or rats to associate neutral olfactory, auditory, or spatial cues with electric shock (Tully and Quinn, 1985).

Many forms of memory decline with age (reviewed in Morrison and Baxter, 2012), including associative memory decline in *Drosophila* (Tamura et al., 2003). *C. elegans* is able to perform a conditioned response after training in which food or starvation is associated with a conditioned stimulus (Hedgecock and Russell, 1975; Saeki et al., 2001; Nuttley et al., 2002; Torayama et al., 2007; Kauffman et al., 2010). Understanding the effects of aging and age-related genetic pathways on associative memory in *C. elegans* can lead to a greater understanding of mechanisms that may regulate associative memory in higher organisms. Age-related experimental data using these paradigms are reviewed in detail below.

### Thermotaxis

*C. elegans* can be conditioned to positively associate a training temperature with the presence of food (Hedgecock and Russell, 1975). After training for four or more hours, worms move to their training temperature in search of food and navigate within this temperature for several hours, a behavior termed *isothermal tracking* (IT) (Mori and Ohshima, 1995; Mohri et al., 2005). Thermotactic ability is assessed using a single-worm assay in which worms are placed on a plate with a radial gradient of temperatures from 17 to 25°C for 90 min, and worm tracks are analyzed to study IT (Mori and Ohshima, 1995; Gomez et al., 2001). Conversely, after starvation on a conditioning plate at a specific temperature, worms avoid that temperature (Mohri et al., 2005; Kodama et al., 2006) or no longer show a preference for that temperature (Chi et al., 2007). Chi et al. (2007) found that worms cultivated at 25°C did not migrate toward 25°C, nor did worms starved at 25°C avoid warmer temperatures. The authors broadly interpreted their results to imply that thermotaxis is not a form of associative learning (Chi et al., 2007). However, Mohri et al. (2005) and Kodama et al. (2006) showed that worms are able to move toward 25°C when cultivated at that temperature, and avoid 25°C when starved at that temperature, suggesting that at least in some training paradigms, worms can form thermal food/starvations associations at 25°C.

One mark of associative behavior is that it can be extinguished by reversing the association. Indeed, after conditioning worms overnight with food at 20°C, Gomez et al. (2001) tested extinction of the temperature-food memory by holding the worms that had previously been cultivated at 20°C on plates without food at 20°C. The number of worms showing IT decreased by 50% after about 7 h had elapsed since training, and returned to pre-conditioned levels by 18 h after training (Gomez et al., 2001). Long-lasting behavioral plasticity as a result of thermotaxis conditioning is modulated by diacylglycerol kinase at the sensory level in the neuron AFD (Biron et al., 2006). Diacylglycerol kinase is also known to regulate long-term reference memory in mice (Shirai et al., 2010). Whether or not thermotaxis meets other criteria of long-term memory, such as the requirements for protein translation, gene transcription, and CREB transcriptional activity, has not yet been tested.

Among worms showing locomotion, there is a moderate but significant decline in IT by day 6 of adulthood (Murakami and Murakami, 2005). By day 12 the fraction of worms with IT after training decreases by half, and is undetectable by day 15 (Murakami and Murakami, 2005). *age-1* Mutants increase IT ability in young and old animals, and have a 210% extension in “high IT” ability (period where more than 75% of worms show IT), but only a 65% lifespan extension compared to wild-type worms (Murakami et al., 2005). Expression of *age-1* in the AIY interneurons restored IT to wild-type levels, but did not affect lifespan, showing that AGE-1 functions directly in AIY neurons to mediate IT, rather than the phenotypic extension being a byproduct of organism-wide lifespan extension (Murakami et al., 2005; Kodama et al., 2006).

*daf-2* and *age-1* mutants have increased IT as compared to wild-type worm when temperature is associated with either food or starvation in young adult worms and with age (Murakami et al., 2005). At both stages, the increase in IT is dependent on *daf-16* (Murakami et al., 2005). The calcium-dependent gene *ncs-1* is essential for IT (Gomez et al., 2001). *ncs-1* Mutants have normal chemotaxis, motility, and thermal avoidance behaviors, but have reduced IT (Gomez et al., 2001). Murakami et al. (2005) tested *ncs-1;daf-2* double mutants to determine whether or not *ncs-1* genetically interacts with the insulin signaling pathway. *ncs-1;daf-2* Mutants have an IT defect compared to wild-type animals, but similar IT to both *ncs-1* and *daf-16* single mutants (Murakami et al., 2005). Though these data suggest that *daf-2* is acting in an *ncs-1-*dependent manner in IT, Murakami et al. (2005) concede that *ncs-1* may be essential for IT in any condition.

In a screen for mutants that do not integrate food conditions with temperature, Mohri et al. (2005) isolated an allele of *ins-1*. While wild-type animals avoid a temperature when it is paired with starvation, *ins-1* mutants move toward their cultivation temperature regardless of the presence of food (Kodama et al., 2006). Kodama et al. (2006) showed that *ins-1* mutants move and respond to feeding states normally, suggesting that they are specifically defective in forming the starvation-temperature association. *daf-2* and *age-1* mutants rescue the *ins-1* phenotype (Kodama et al., 2006). To analyze how *ins-1* regulates IT, Kodama et al. (2006) analyzed calcium dynamics in the AIZ interneuron, which is essential for thermotaxis (Mori and Ohshima, 1995). In the presence of food, intracellular calcium is increased at higher temperatures and decreased at lower temperatures in wild-type animals (Kodama et al., 2006). The response of AIZ to temperature is dampened in starvation conditions in wild-type animals (Kodama et al., 2006). In *ins-1* mutants, the response of AIZ to temperature is never dampened, suggesting that *ins-1* regulates the integration of the starvation-temperature association (Kodama et al., 2006).

At both days 1 and 9 of adulthood, *eat-2* mutants increase IT when associating food, but not starvation, with a specific temperature, indicating that CR affects food-temperature association (Murakami et al., 2005). The mitochondrial mutant *clk-1* also has increased IT at days 1 and 9 of adulthood (Murakami et al., 2005). Murakami and Murakami (2005) found that mutants with lower oxidative stress (*clk-1* and *isp-1*), increased IT in young adults, while those with high levels of oxidative stress (*gas-1* and *mev-1*), decreased IT. Treating *mev-1* mutants with the antioxidant lipoic acid partially rescued IT ability (Murakami and Murakami, 2005). *C. elegans* lifespan is shorter after long-term cultivation at higher temperatures (Klass, 1977). Thermotaxis associative learning assays show that *C. elegans* respond to even short-term changes in temperature and that these responses are temperature specific and also influenced by aging pathways.

### Salt chemotaxis learning

*C. elegans* can associate salt concentration with starvation conditions (Saeki et al., 2001). Untrained worms are attracted to 100 mM salt, and this attraction is increased when worms are starved in buffer alone (Bargmann and Horvitz, 1991; Tomioka et al., 2006; Kano et al., 2008). Animals trained to associate salt with starvation remember this association for up to 60 min, forming a stable short-term memory (Tomioka et al., 2006; Kano et al., 2008). Whether salt learning and memory change with age has not yet been tested.

After starvation in the presence of salt, the long-lived insulin signaling pathway mutants *daf-2*, *age-1*, *pdk-1*, and *akt-1* are still attracted to salt (Tomioka et al., 2006), suggesting that they are defective in forming the starvation-salt association. Interestingly, loss of *daf-16* does not rescue the *daf-2* or *age-1* mutant phenotypes, suggesting that the defect of *daf-2* in salt learning is independent of *daf-16* (Tomioka et al., 2006). Similar to thermotaxis, INS-1 was identified as the insulin-like peptide that may regulate this association, as *ins-1* mutants show neither an avoidance of salt after starvation training nor an increased attraction to salt after starvation in the absence of salt (Tomioka et al., 2006). Salt learning is rescued by expression of *age-1* or *daf-2* in the ASER neuron, and by *ins-1* expression in AIA, suggesting that feedback between the AIA interneurons and the ASER sensory neuron results in associative learning that is mediated by INS-1 (Tomioka et al., 2006).

After conditioning without food in the presence of salt, the ASER neuron exhibits a sharp increase in calcium activity and a decrease in synaptic release following a down-step in salt concentration as compared to animals trained without salt (Oda et al., 2011). In the insulin signaling mutants *daf-2*, *ins-1*, and *age-1*, calcium signaling and synaptic release after salt conditioning are indistinguishable from mock-trained animals (Oda et al., 2011). Since insulin signaling in salt learning is active in ASER, this pathway may modulate activity and vesicle release specifically in ASER to reduce salt attraction. What salt response looks like with age and how insulin signaling regulates changes in calcium activity and synaptic function after salt learning remain to be tested.

### Positive olfactory associative learning and memory

Along with thermotaxis and salt learning, *C. elegans* can learn to associate an odor with food. Torayama et al. (2007) showed that after a single exposure to food and butanone, worms have a 20% increased chemotaxis index to butanone compared to naïve animals, termed “butanone enhancement” (Torayama et al., 2007). After conditioning worms for 90 min, this enhancement lasted 4 h if they were starved after conditioning, but only 1 h if they were fed afterward (Torayama et al., 2007). The susceptibility of butanone enhancement to age-related decline and in longevity mutants has not been tested. *ins-1* Mutants have normal butanone enhancement but cannot properly associate butanone with starvation (Lin et al., 2010).

In a different food/butanone training paradigm, Kauffman et al. (2010) found that worms that are briefly starved and then trained to associate butanone and food increase their chemotaxis toward butanone by 60%. Using this paradigm, Kauffman et al. (2010) designed both massed- and spaced-training paradigms that result in short-term associative memory (STAM) and long-term associative memory (LTAM), respectively. Briefly, these assays involve a short starvation in buffer, followed by conditioning with food and butanone either once (massed training) or seven times (spaced-training) (Kauffman et al., 2010). After training, worms are held on food without butanone (to allow them to forget the association) and are tested using a standard attraction chemotaxis assay (Troemel et al., 1997). STAM declines within 2 h, but LTAM lasts between 16 and 24 h after training (Kauffman et al., 2010). LTAM is dependent on transcription, translation, and CREB activity (Kauffman et al., 2010), factors that have been shown in other organisms, such as flies, *Aplysia*, and mice, to be required for long-term memory (reviewed in Silva et al., 1998).

To determine how aging affects associative memory in *C. elegans*, Kauffman et al. examined motility, chemotaxis, massed learning, spaced learning, and 16 h long-term memory for the first week of adulthood. While movement and chemotactic ability were maintained, 16 h LTAM decreased by day 2 of adulthood, and was undetectable by day 5 (Kauffman et al., 2010). Massed learning declined soon thereafter, while 7× spaced learning was undiminished at day 3, but declined by day 7 of adulthood (Kauffman et al., 2010). These cognitive declines precede age-related changes in chemotaxis, IT (Murakami and Murakami, 2005), habituation (Beck and Rankin, 1993), and motility, suggesting that 16 h LTAM and massed learning are most sensitive to age-related changes (Kauffman et al., 2010). Interestingly, this decline in cognitive function also occurs earlier than observable age-related morphological decline in neurons and muscles (Herndon et al., 2002; Pan et al., 2011; Tank et al., 2011; Toth et al., 2012).

Kauffman et al. (2010) next tested whether memory of positive olfactory conditioning in *C. elegans* is controlled by known longevity pathways, and found that (1) *daf-2* mutants remember significantly longer than do wild-type animals on the first day of adulthood; (2) *daf-2* mutant STAM lasts over three times as long as wild-type, and (3) *daf-2* LTAM lasts longer than 40 h after training. The extension of learning and memory in *daf-2* mutants requires the DAF-16 transcription factor, as *daf-16* mutants have defects in massed learning, STAM, and LTAM. *daf-2* Mutants are also able to establish a 16-h long-term association after only five training sessions as compared to seven sessions for wild-type, although their massed learning rate is similar to wild-type’s. To determine whether all longevity pathways have similar effects on learning and memory, Kauffman et al. (2010) also examined these behaviors in the CR model, *eat-2*. Unlike *daf-2*, *eat-2* mutants have normal learning and short-term memory, indicating that the feeding conditions used for training are not compromised in the *eat-2* mutant, but that the STAM extension observed in *daf-2* mutants is not generalizable to all longevity mutants. Additionally, *eat-2’s* long-term memory is only 60% that of wild-type worms, suggesting that CR somehow impairs formation of the memory of the association between food and butanone, although LTAM can be restored by increasing training to 10 cycles (Kauffman et al., 2010). *eat-2’s* Defective LTAM phenotypes are dependent on the *pha-4* transcription factor (Kauffman et al., 2010), which is also required for *eat-2’s* longevity effects (Panowski et al., 2007). Feeding *eat-2* mutants a smaller, easier to digest bacteria, *Comamonas* sp., rescues their small body size (Avery and Shtonda, 2003) and reverses *eat-2’s* lifespan extension (Kauffman et al., 2010), showing that *Comamonas* sp. feeding “undoes” CR. Kauffman et al. (2010) found that feeding *eat-2* mutants *Comamonas* sp. also rescued *eat-2’s* LTAM defect. Together, these results suggest that CR, rather than the acetylcholine receptor mutation that causes the defective pharyngeal pumping in *eat-2* worms, is responsible for *eat-2’s* memory defects (Kauffman et al., 2010). Therefore, different longevity pathways have different effects on learning and memory early in adulthood.

To determine the effects of the IIS and CR pathways on maintenance with age, Kauffman et al. tested the worms’ performance on day 4 of adulthood. While *eat-2* animals have reduced 16 h memory on the first day of adulthood compared to wild-type worms, their memory ability is maintained at least until day 4 of adulthood and this maintenance also requires *pha-4* (Kauffman et al., 2010). By contrast, *daf-2* learning is maintained better than wild-type with age, but 16 h long-term memory at day 4 of adulthood is entirely abrogated, as it is in wild-type worms (Kauffman et al., 2010).

To resolve these seemingly disparate results, Kauffman et al. (2010) tested the cAMP response element binding protein (CREB) transcription factor, which is required for long-term memory in all organisms tested (reviewed in Silva et al., 1998) including *C. elegans* (Kauffman et al., 2010). Kauffman et al. examined whether CREB expression and activated protein levels correlated with LTAM retention in young, old, *daf-2*, and *eat-2* worms. Along with an increase in LTAM, *daf-2* day 1 adults have higher levels of CREB protein than do day 1 wild-type worms (Kauffman et al., 2010). CREB levels and activity in both *daf-2* and wild-type worms decrease with age, as does long-term memory (Kauffman et al., 2010). Conversely, *eat-2* worms have lower levels of CREB and defective memory at day 1 of adulthood, but both CREB levels and day 1 adult long-term memory level are maintained with age (Kauffman et al., 2010). Therefore, the differential effects of the insulin and dietary restriction pathways with age could be attributable to their differences in CREB expression and activity levels, and CREB levels are predictive of memory performance.

These data agree with previous findings in mammals that show that along with decreased cognitive function, total CREB and CREB activity levels decline with age (Asanuma et al., 1996; Brightwell et al., 2004; Porte et al., 2008) and long-term memory can be rescued by over-expression of CREB in the hippocampus (Mouravlev et al., 2006). Therefore, the mechanisms required for CREB regulation of long-term memory in *C. elegans* may be conserved in higher organisms. Indeed, increased memory and neuronal plasticity in mice following CR requires CREB (Fusco et al., 2012); similar studies in IIS-reduced conditions in mice would be interesting to examine. Furthermore, the downstream targets of CREB that become activated upon memory training are presumably the cellular components that actually enable memory function, and therefore are important to identify. *C. elegans* presents a tractable system to identify genome-wide memory-specific transcriptional targets of CREB, which can then be compared with CREB overexpression data (Barco et al., 2002) and *Drosophila* memory studies (Dubnau et al., 2003).

### Olfactory avoidance learning

After a single exposure to an aversive concentration of benzaldehyde and starvation, *C. elegans* avoids an attractive concentration of the same odor (Nuttley et al., 2002). Worms are starved in the presence of benzaldehyde and then tested for learning using an attraction chemotaxis assay (Troemel et al., 1997; Nuttley et al., 2002). This behavior is referred to as benzaldehyde-starvation associative plasticity. Whether this behavior is maintained or changes with age has not been reported. As in salt learning, *ins-1*, *daf-2*, and *age-1* mutants lack the ability to fully associate benzaldehyde and starvation (Lin et al., 2010). Rescuing *ins-1* only in adulthood or in AIA and ASI, two sets of interneurons, rescues the learning defect in these mutants (Lin et al., 2010). Rescuing *age-1* specifically in AWC, the neurons that sense benzaldehyde, rescues the learning defect.

Whether *daf-2* primarily regulates memory recall or formation in massed benzaldehyde-starvation associative plasticity was addressed next (Lin et al., 2010). The *daf-2(e1370)* allele has temperature-sensitive learning phenotypes in both salt learning and olfactory avoidance learning (Tomioka et al., 2006; Lin et al., 2010). When conditioned and tested at 15°C, *daf-2’s* olfactory avoidance learning is normal, but when conditioned and tested at 23°C, its learning is completely abrogated (Lin et al., 2010). Lin et al. (2010) found that training *daf-2* mutants at the restrictive temperature (23°C) resulted in memory formation if testing was done at the permissive temperature (15°C). Conversely, training at 15°C then testing at the 23°C resulted in a lack of benzaldehyde-starvation associative learning (Lin et al., 2010). Since testing *daf-2* at the restrictive temperature after benzaldehyde-starvation training at either temperature shows a total loss of olfactory avoidance learning, but testing at the permissive temperature after training at the restrictive temperature shows only a small loss, *daf-2* may function primarily in recall of olfactory avoidance learning. This agrees with the Kauffman et al. (2010) finding that *daf-2* worms learn a butanone-food association at the same rate as wild-type, but retain a short-term memory of this association longer, and thus primarily affects recall, at least in a massed training paradigm.

### Non-associative learning and memory

Age-related decline and the effects of longevity pathways have been studied in some forms of associative learning and memory as reviewed above. However, less is known about the effect of aging on non-associative forms of memory. *C. elegans* can adapt to, be sensitized to, habituated to, or dishabituated to stimuli (Rankin et al., 1990; Colbert and Bargmann, 1995) as can higher organisms such as *Drosophila*, rats, and humans (Engen et al., 1963; Thompson and Spencer, 1966; Fox, 1979). Naïve response to tap and habituation change with age (Rankin et al., 1990; Beck and Rankin, 1993). Day 9 adult worms respond to tap with smaller reversals, and recover from habituation more slowly than do days 1 or 4 adult worms (Beck and Rankin, 1993). Like LTAM (Kauffman et al., 2010), early adulthood long-term memory of habituation requires CREB (Timbers and Rankin, 2011). It remains to be seen whether or not these age-related changes are regulated by longevity pathways.

*C. elegans* can also be trained to disregard an inherently attractive odor by long-term exposure to that odor, a form of olfactory adaptation (Colbert and Bargmann, 1995). Currently, age-related adaptation has not been tested. Murakami et al. (2005) found that *daf-2* mutants have increased adaptation to benzaldehyde. However, since worms are starved in the presence of benzaldehyde in this assay and learning is blocked by conditioning with food, they may in fact be forming a negative associative memory instead of adapting (Nuttley et al., 2002; Pereira and van der Kooy, 2012). Chalasani et al. (2010) found that *ins-1(nr2091)* mutants cannot adapt to isoamyl alcohol when starved. By contrast, Pereira and van der Kooy (2012) found that both *daf-2* and *ins-1(nj32)* adapt normally after conditioning with isoamyl alcohol in the presence or absence of food.

## Discussion

### Learning and memory decline with age

**Figure 1:**
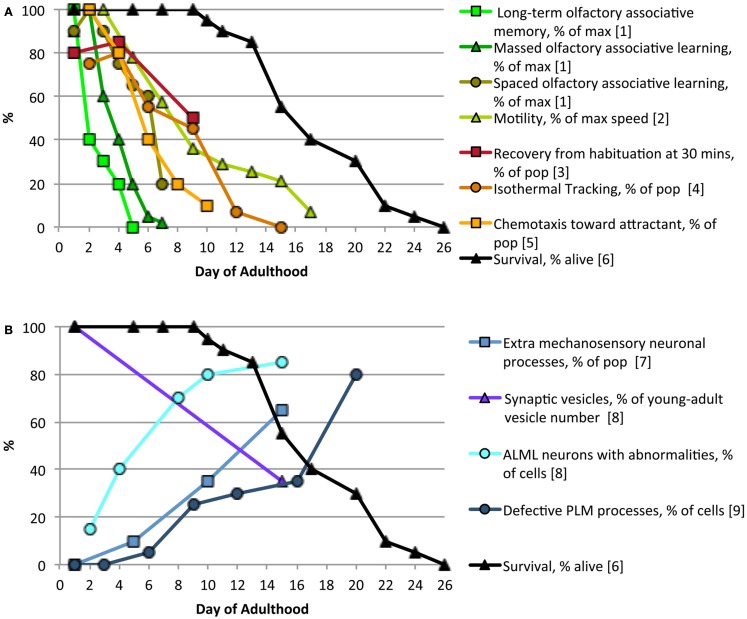
**Age-related changes in neuron morphology and behavior**. Behaviors based on sensory perception **(A)** decline rapidly with age, preceding accumulation of most identified neuronal defects **(B)**, long before worms start to die [solid black line in **(A,B)**]. Complex sensory behaviors, such as long-term associative memory, decline prior to mechanosensory behaviors, such as locomotion. References: (1) Kauffman et al., 2010, (2) Hsu et al., 2009, (3) Beck and Rankin, 1993, (4) Murakami and Murakami, 2005, (5) Glenn et al., 2004, (6) Luo et al., 2009, (7) Tank et al., 2011, (8) Toth et al., 2012, (9) Pan et al., 2011.

Multiple forms of learning and memory decline with age in *C. elegans* as they do in *Drosophila*, mice, and humans (Bach et al., 1999; Tamura et al., 2003; Murakami and Murakami, 2005; Doty, 2009; Kauffman et al., 2010). In *C. elegans* cognitive decline occurs as early as day 2 of adulthood, when 16 h LTAM of an odor/food pairing is already significantly decreased (Kauffman et al., 2010). Figure 1 illustrates age-related declines in learning and memory (Figure 1A) as well as morphological changes in neurons with age (Figure 1B). The decline in IT could be explained by increased neuronal outgrowths and decreased synaptic vesicle density, but several types of learning and memory decline far before neurons exhibit these gross morphological changes. While changes in sensory neuron and interneuron subcellular structures may be responsible for early behavioral declines, there may be an earlier decline in neuronal signaling, due to changes in learning and memory gene expression levels. Indeed, Kauffman et al. (2010) found that reduction in CREB expression levels and activity with age correlate with reduced LTAM ability and occur far before structural and signaling changes are observed. Further analysis of neuronal activity using calcium indicators in multiple neurons could determine the full extent and timing of sensory decline with age.

### Longevity pathways, learning and memory

Mutants in three longevity pathways have been tested in multiple learning paradigms. Phenotypes of different mutants are listed in Table 1. *eat-2* CR mutants have enhanced associative thermotaxis (Murakami et al., 2005) and a defect in LTAM, but have stable long-term memory in the first 4 days of adulthood, unlike wild-type worms (Murakami et al., 2005; Kauffman et al., 2010). *eat-2* Mutants have normal neuronal morphology with age (Tank et al., 2011). However, *eat-2* mutants have lower but stable CREB protein levels with age instead of decreasing levels as in wild-type worms, which may contribute to the stable LTAM phenotype (Kauffman et al., 2010). How CR enhances the thermotaxis/food association or affects CREB levels in this paradigm is unknown. Interestingly, short-term CR in older people was recently shown to improve verbal memory (recall of a word list after 30 min) compared to controls (Witte et al., 2009). Increased insulin in humans through intranasal injection also improves verbal memory (Benedict et al., 2007).

**Table 1:** **Longevity pathway mutant phenotypes compared to wild-type worms**.

Phenotype with respect to wild-type	*ins-1*	*daf-2*	*age-1*	*daf-16*	*eat-2*	*clk-1*
Neuron integrity (Pan et al., 2011; Tank et al., 2011)	n/d	+	n/d	−	No change	+
Positive associative thermotaxis (Murakami and Murakami, 2005; Murakami et al., 2005)	No change	+	+	−	+	+
Negative associative thermotaxis (Murakami and Murakami, 2005; Murakami et al., 2005)	−	+	+	−	No change	No change
Salt learning (Tomioka et al., 2006)	−	−	−	−	Naïve defect	n/d
Olfactory adaptation (Chalasani et al., 2010, Pereira and van der Kooy, 2012)	−	+	n/d	n/d	n/d	n/d
Massed positive olfactory learning (Kauffman et al., 2010; Lin et al., 2010)	No change	No change	n/d	−	No change	n/d
Short-term associative memory (Kauffman et al., 2010)	n/d	+	n/d	−	No change	n/d
Spaced olfactory learning (Kauffman et al., 2010)	n/d	No change	n/d	−	No change	n/d
Long-term associative memory (Kauffman et al., 2010)	n/d	+	n/d	−	−	n/d
Olfactory avoidance learning (Lin et al., 2010)	−	−	−	n/d	n/d	n/d

*Mutants listed have been tested in at least two learning and memory paradigms reviewed here, or for neuron aging phenotypes and one learning and memory paradigm reviewed here*.

*(+), Aqua: increased compared to wild-type. (−), Yellow: defective compared to wild-type. (no change) The same as wild-type. (n/d) Not determined. (naïve defect) Chemotaxis defect before training*.

The *clk-1* electron transport chain mutant has enhanced cellular integrity with age and enhanced positive thermotaxis (Murakami and Murakami, 2005; Tank et al., 2011). The increased thermotaxis is likely due to *clk-1*’s slower metabolism, as *isp-1* also has increased thermotaxis, but *mev-1* and *gas-1*, which have increased mitochondrial metabolic rates, display decreased thermotaxis (Murakami and Murakami, 2005; Murakami et al., 2005). While these data support the theory that ROS levels regulate age-related decline, whether or not mitochondrial metabolism or ROS is directly involved in learning and memory, or if the learning phenotype is instead a byproduct of the effects of slowed aging, is currently unknown.

The requirement of insulin signaling has been tested in multiple associative learning and memory paradigms. *daf-2* Mutants appear to differentially regulate different learning paradigms, as they have enhanced thermotaxis (Murakami et al., 2005), long-term and short-term positive associative learning and memory (Kauffman et al., 2010), and improved neuron integrity (Pan et al., 2011; Tank et al., 2011; Toth et al., 2012), but are deficient in salt learning (Tomioka et al., 2006) and olfactory avoidance learning (Lin et al., 2010). The inability of *daf-2* mutants to perform the two latter forms of learning, which rely on negative associations, perhaps indicates that *daf-2* mutants are unable to form negative associations as readily as wild-type. Since *daf-2* mutants are more resistant to stresses such as heat, paraquat, and starvation in low bacteria concentrations (Houthoofd et al., 2003), they may need increased conditioning time or more extreme stress conditions to form negative associations.

Unlike mitochondrial metabolism, it is clear that insulin signaling regulates learning separately from longevity, since neuron-specific rescue of *daf-2*, *age-1*, or *daf-16* can rescue learning phenotypes without restoring normal lifespan (Murakami et al., 2005; Kodama et al., 2006; Tomioka et al., 2006; Lin et al., 2010). This may simply be a result of the cell autonomous nature of neuronal phenotypes, as opposed to the system-wide nature of longevity and dauer regulation (Apfeld and Kenyon, 1998). *daf-2* Mutants have higher CREB levels and increased long-term associative olfactory memory as well as extended learning and STAM with age (Kauffman et al., 2010). For thermotaxis and positive associative olfactory learning and memory, it may be interesting to test downstream targets of *daf-16* for learning and memory effects. However, *daf-2* mutant response to salt learning is independent of *daf-16* and may rely on different *daf-2* signaling output. *daf-2* Mutants (as compared to *daf-16;daf-2* mutants) expressed lower levels of the guanylyl cyclases *gcy-18* and *gcy-6* (Murphy et al., 2003). As these genes are normally expressed in neurons required for thermotaxis (AFD) and salt learning (ASE), respectively, some of *daf-2’s* defects may be due to altered signaling within those neurons.

*ins-1* May be the cue for integration of a starvation-stimulus association, but not a food-stimulus association. *ins-1* Mutants are still attracted to odors, salt, and temperature even after starvation (Mohri et al., 2005; Tomioka et al., 2006; Lin et al., 2010). Rescuing *ins-1* expression in the AIA interneuron restores both salt and olfactory avoidance learning (Tomioka et al., 2006; Lin et al., 2010). This is especially intriguing since rescue of *daf-2* or *age-1* in the sensory neurons ASER or AWC restores normal behavior in olfactory avoidance learning and salt learning, respectively (Tomioka et al., 2006; Lin et al., 2010). These data inform a model in which INS-1 is secreted by AIA after association of starvation with a stimulus, and acts in a feedback loop to regulate insulin signaling in the sensory cells (Chalasani et al., 2010). The mechanism of INS-1 secretion in AIA and the downstream effects on insulin signaling in the sensory neurons are unknown, though Chalasani et al. (2010) found that *ins-1* does not require *daf-2* for regulation of turning behavior, suggesting that INS-1 can act through a different and as yet unidentified receptor. This would be an interesting alternative to the “40 insulins/one insulin receptor” model of insulin signaling in *C. elegans*. Because *daf-2*, *age-1*, and *ins-1* mutants have defective AIZ interneuron responses to starvation and thermotaxis, *ins-1* may also regulate insulin signaling in downstream interneurons (Kodama et al., 2006).

## Conclusion

*C. elegans* is an established organism in the field of aging and longevity, as well as a model for complex behaviors, such as learning and memory. While many organisms show age-related declines in learning and memory, few molecular mechanisms that regulate these processes have been identified. Since longevity pathways actively regulate learning and memory declines and morphological changes in neurons, *C. elegans* will be an ideal system to identify molecular mechanisms that regulate such declines. Our understanding of both normal aging and neurodegenerative disease-related decreases in learning and memory at a cellular, synaptic, and molecular level will be aided by further *C. elegans* investigations at the intersection of aging, longevity, and neurobiology.

## Conflict of Interest Statement

The authors declare that the research was conducted in the absence of any commercial or financial relationships that could be construed as a potential conflict of interest.
